# The prospects and limitations of liquid biopsy utilization for clinical practice in Taiwan

**DOI:** 10.1016/j.jlb.2025.100290

**Published:** 2025-03-06

**Authors:** Huei-Ying Li, Chun-Chuan Chang, Yu-Hsuan Yang, Chi-Yuan Yao, Jason Chia-Hsun Hsieh, Shao-Hsuan Chang

**Affiliations:** a Medical Microbiota Center of the First Core Laboratory, National Taiwan University, Taipei, Taiwan; b Professional Master's Program of Biotechnology Management, National Taiwan University, Taipei, Taiwan; c Graduate Institute of Medical Genomics and Proteomics, National Taiwan University, Taipei, Taiwan; d Department of Laboratory Medicine, National Taiwan University Hospital, Taipei, Taiwan; e Division of Oncology, Department of Internal Medicine, Linkou Chang Gung Memorial Hospital, Taoyuan, Taiwan; f College of Medicine, Chang Gung University, Taoyuan, Taiwan; g Department of Biomedical Engineering, Chang Gung University, Taoyuan, Taiwan

**Keywords:** Liquid biopsy, Cancer diagnosis, Minimal residual disease (MRD), National health insurance, Clinical adoption

## Abstract

**Objective:**

Liquid biopsy is a promising, non-invasive diagnostic tool for cancer, offering rapid and cost-effective genomic analysis. It provides a less invasive alternative to traditional tissue biopsies, with potential benefits in monitoring disease progression and detecting minimal residual disease (MRD). However, its clinical integration faces challenges, including utility assessment and workflow adaptation. This study evaluates the value of liquid biopsy in Taiwan from a clinical physician's perspective.

**Methods:**

A survey was conducted with 16 physicians specializing in thoracic medicine and hematologic oncology. Participants responded to a 5-point Likert scale to evaluate the timing of liquid biopsy adoption, willingness to incorporate it into clinical practice, and agreement on its role in managing specific clinical conditions.

**Results:**

Forty percent of physicians preferred liquid biopsy when tissue samples were unavailable. The inclusion of liquid biopsy under National Health Insurance (NHI) was a key factor in its adoption. Hematologic oncologists showed a stronger preference for liquid biopsy, particularly for MRD testing, compared to their counterparts in thoracic medicine (hematologic oncology vs. thoracic medicine: 4.2 ± 0.83 vs. 3.1 ± 0.60; p value = 0.01). Younger physicians valued turnaround time, while senior physicians prioritized test brand, with a focus on report speed.

**Conclusion:**

Physicians are generally less inclined to replace tissue biopsies with liquid biopsy, but hematologic oncologists show more flexibility. Test brand plays a role in physician decision-making, and the inclusion of liquid biopsy under NHI coverage is vital for its broader adoption in Taiwan.

## 1 Introduction

Liquid biopsy is an emerging, non-invasive diagnostic tool for cancer that facilitates the detection of tumor-related biomarkers in bodily fluids, typically blood samples. It is primarily utilized for analyzing circulating tumor cells (CTCs) or circulating tumor DNA (ctDNA), which are released from the primary tumor into the bloodstream [[1], [2], [3]]. Functional analyses of CTCs can provide valuable insights into their ability to replicate at metastatic sites, persist in circulation, and invade surrounding tissues [4]. Meanwhile, ctDNA consists of small fragments of tumor DNA released into the bloodstream from apoptotic or necrotic tumor cells. It has demonstrated potential for early cancer detection by identifying tumor-specific genetic changes [5]. Together, these biomarkers offer critical information about tumor presence, progression, and response to therapy.

Compared to traditional tissue biopsies, liquid biopsy is a minimally invasive approach for detecting circulating tumor DNA (ctDNA) using advanced technologies, such as next-generation sequencing (NGS), real-time PCR (RT-PCR), droplet digital PCR (ddPCR), and mass spectrometry. Among these, NGS-based techniques including targeted sequencing, whole-exome sequencing, and whole-genome sequencing allow for the comprehensive analysis of tumor mutations and genomic alterations, with a sensitivity of up to 0.01 % variant allele frequency (VAF) [6]. Liquid biopsy also enables continuous monitoring, which is especially valuable for patients who are difficult to biopsy or those requiring repeated sampling during treatment [7,8]. Quantitative analysis of tumor-specific mutations in ctDNA, such as single nucleotide variants in *KRAS*, *NRAS*, *PIK3CA*, *BRAF*, and *EGFR*, demonstrated over 80 % concordance with tumor tissue in patients with colorectal, lung, and breast cancers [9]. NGS is the most commonly used approach, as it facilitates large-scale, parallel sequencing of small DNA fragments based on known tumor mutations. However, for samples with limited availability, ddPCR provides an alternative with high sensitivity, detecting nucleotide mutations in ctDNA at concentrations as low as 0.01 % while precisely quantifying DNA molecules. ddPCR has been applied to quantify oncogene mutations in ctDNA, such as *PIK3CA* mutations in early-stage breast cancer, achieving a sensitivity of 0.01 % for detecting each of the three *PIK3CA* hotspot mutations and a specificity exceeding 99 % compared to tumor tissue [10]. Therefore, while NGS is well-suited for comprehensive multi-gene analysis, ddPCR excels in the high-sensitivity detection of low-frequency mutations. Additionally, ddPCR offers advantages in detection speed and cost compared to NGS, making it a more practical option for patients with financial constraints or those requiring urgent first-line (1 L) treatment. In summary, NGS remains the preferred choice for clinical applications due to its broad genetic detection capability and high sensitivity and specificity. Meanwhile, ddPCR, with its rapid turnaround time (TAT), lower cost, and high sensitivity, serves as an optimal option for patients with limited financial resources or those needing rapid clinical decision-making. The complementary use of these two technologies enables the optimization of diagnostic and therapeutic strategies tailored to different clinical needs [11,12].

As the clinical utility of liquid biopsy continues to expand, ctDNA analysis is increasingly being integrated into treatment decision-making for NSCLC. In recent years, the role of multi-gene NGS-based plasma ctDNA analysis has been further emphasized in clinical guidelines, marking a significant shift from single-gene testing (e.g., *EGFR* mutation) to a broader and more comprehensive approach for advanced NSCLC patients. This move reflects the increasing complexity of molecular targets in NSCLC, as well as the growing evidence suggesting that NGS is more cost-effective than traditional sequential or hotspot-based platforms. However, challenges regarding the availability and reimbursement of NGS technology remain a major concern in many regions, particularly as the number of actionable mutations continues to rise. The dynamic nature of ctDNA allows for serial monitoring, providing a non-invasive means to assess clonal evolution and treatment efficacy over time. In this context, multi-gene NGS has been recommended by leading organizations such as the International Association for the Study of Lung Cancer (IASLC) and the European Society for Medical Oncology (ESMO) for molecular profiling in advanced NSCLC [13]. Additionally, the current ESMO Clinical Practice Guidelines recommend comprehensive genotyping for metastatic NSCLC and non-squamous NSCLC patients to guide targeted therapy decisions. For treatment naive NSCLC, ctDNA is served as a complementary or alternative tool to tissue-based NGS for biomarker assessment [14]. The ability of ctDNA assays to detect resistance mutations in real time has significantly improved therapeutic strategies, particularly in guiding the use of targeted therapies. For instance, osimertinib has become the standard of care (SOC) for patients with acquired resistance due to the *EGFR* T790M mutation, demonstrating the feasibility of a plasma-first approach to minimize the need for invasive tissue biopsies.

In addition to providing insights into genetic alterations, one of the most significant advantages of liquid biopsy is its ability to detect minimal residual disease (MRD), a key indicator of cancer recurrence that traditional imaging techniques often fail to identify [[15], [16], [17]]. MRD refers to the small number of cancer cells that may remain in a patient's body after treatment, which can potentially lead to relapse [4,18]. Liquid biopsy is particularly suited for monitoring MRD because it can identify even the smallest amounts of tumor-related genetic material in bodily fluids [19]. This makes liquid biopsy a powerful tool for assessing treatment efficacy, detecting early signs of relapse, and tailoring personalized treatment plans to prevent recurrence.

While liquid biopsy holds significant promise for detecting MRD, it shares some of the same challenges as early cancer detection, particularly in cases with low tumor burden [20]. In such situations, the levels of ctDNA or CTCs may be insufficient for accurate detection. This is evident in cancers such as early-stage lung cancer, pancreatic cancer, colorectal cancer, ovarian cancer, and localized prostate cancer, where low tumor burden may limit the sensitivity and effectiveness of liquid biopsy for monitoring disease progression or detecting MRD [11,21]. Although liquid biopsy has demonstrated a detection rate of up to 80 % in early-stage non-small cell lung cancer (NSCLC), the National Health Insurance (NHI) system in Taiwan currently reimburses EGFR mutations detected through tissue biopsy, rather than those identified via liquid biopsy. This is likely due to concerns about the lower concentration of ctDNA in the blood, which raises questions about the sensitivity of liquid biopsy in clinical settings.

These challenges have led to ongoing discussions regarding the clinical utility of liquid biopsy and its integration into existing diagnostic workflows. Therefore, to better understand its potential, this study aims to explore clinicians' perspectives on the adoption, utility, and challenges of liquid biopsy in routine practice. By surveying and comparing the experiences of specialists in hematologic oncology and thoracic medicine, we aim to assess their views on how liquid biopsy enhances diagnostic accuracy, improves patient monitoring, and explores the conditions under which they would be willing to adopt it in clinical practice. This comparative approach will provide valuable insights into how liquid biopsy is perceived across different specialties, helping to identify both clinical barriers and opportunities for broader adoption in cancer care.

## 2 Materials and methods

### 2.1 Questionnaires and survey design

This study employed a combination of questionnaires and brief interviews to gather insights from clinicians regarding their perspectives on the adoption of liquid biopsy in clinical practice. The primary aim of the questionnaire was to assess the factors influencing physicians' willingness to adopt liquid biopsy and to identify the obstacles that may hinder its broader implementation. Specifically, for solid tumors and hematological cancers, the study focused on whether physicians consider liquid biopsy more effective than traditional tissue biopsy in monitoring treatment response and tracking prognosis. Additionally, the study examined the factors that influence the timing of adoption for large gene panels in clinical settings.

The questionnaire consisted of 10 main questions (Supplementary Table 1) and was tailored to both specialties (hematologic oncology and thoracic medicine), with a particular focus on lymphoma and NSCLC. The survey assessed physicians' views on potential of liquid biopsy, covering the areas of: I) Diagnosing cancer (including first-line and second-line treatment; II) Monitoring disease progression and treatment response; III) Evaluating the effectiveness of large gene panel tests; IV) Detecting MRD and tumor heterogeneity; V) Replacing traditional diagnostic methods (such as blood smears and tissue biopsies).

A total of 16 responses were collected from clinical physicians with experience in utilizing liquid biopsy in their practice. These included 8 hematologic oncologists and 8 thoracic medicine specialists, all of whom practice at medical centers and are familiar with the clinical applications of liquid biopsy tests. The questions were structured as follows: Question 1: Both hematology oncology and thoracic medicine physicians were asked to identify when they would consider using liquid biopsy for their respective cancer types, focusing on early detection, first-line and second-line treatment, tissue samples inaccessible and disease monitoring. Question 2: Factors influencing adoption (such as NHI reimbursement, regulatory approval, test sensitivity, accuracy, price, brand, and turnaround time) are consistent between the two specialties. Question 3–5: Both specialties were asked about their willingness to use liquid biopsy in place of traditional diagnostic tools (blood smears or tissue biopsies) once clinical efficacy has been validated. Questions 6–9: Both groups were asked if they agree that liquid biopsy could be more effective in detecting tumor heterogeneity, monitoring treatment response, detecting drug resistance, and identifying MRD. Question 10: Both groups are asked under which circumstances they would consider using a large gene panel for liquid biopsy. This question remains open-ended in both surveys, allowing for more nuanced, condition-specific responses.

### 2.2 Statistical analysis

For quantitative analysis, a Likert scale was employed, with responses rated on a five-point scale to gauge physicians' agreement or willingness to adopt liquid biopsy. The scale was as follows: 1 = Strongly Disagree/Very Unwilling; 2 = Disagree/Unwilling; 3 = Neutral; 4 = Agree/Willing; 5 = Strongly Agree/Very Willing. Descriptive statistics were calculated for each group to summarize the data. Mean and standard deviation (SD) were reported for each group to provide an overview of the physicians' attitudes toward liquid biopsy. Higher scores reflected a more favorable attitude toward liquid biopsy. The total scores were summed and averaged to calculate the overall level of support for liquid biopsy.

To assess differences between the two groups of physicians (by specialty and by experience), Mann-Whitney *U* test was applied. This non-parametric test was used to compare the distribution of responses between the two groups, as the data were ordinal and did not assume normality. All statistical analyses were performed using Python. A significance level of 0.05 was used for all tests, indicating that the two groups had significantly different views on the adoption of liquid biopsy.

## 3 Results

### 3.1 The adoption of liquid biopsy

**Fig. 1:**
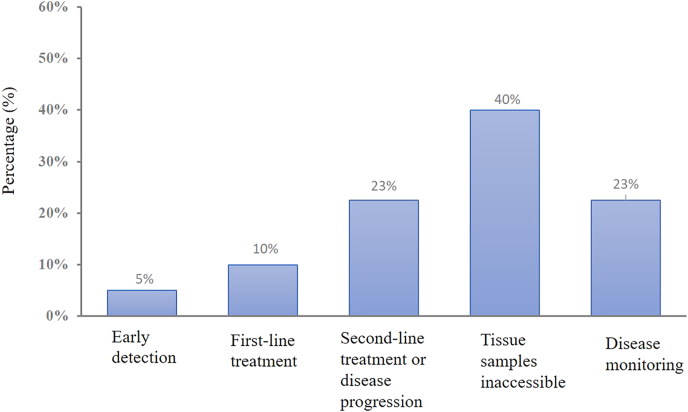
The proportion of adoption of liquid biopsy.

**Fig. 2:**
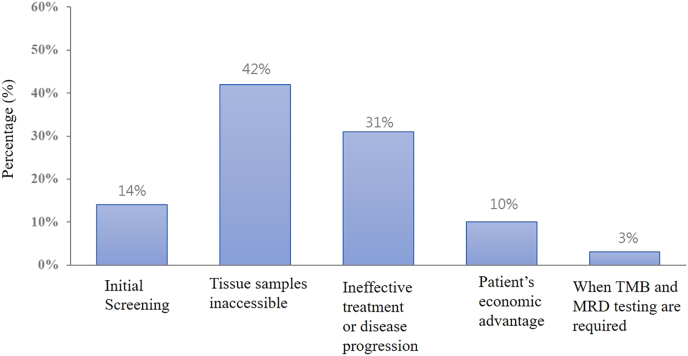
The proportion of adoption of the large gene panel.

The survey results illustrated various scenarios in which liquid biopsy was adopted in the management of solid tumors, categorized into five key areas (Fig. 1). Firstly, the application of liquid biopsy in early detection was relatively limited, likely due to the ongoing need for improvements in both the popularity and accuracy of early detection methods. However, the use of liquid biopsy increases during the first-line treatment, highlighting its significance in the diagnostic process. As the disease progresses, the adoption further raised during second-line treatment or disease exacerbation, reflecting its value in monitoring disease progression. Notably, when tissue samples were inaccessible, the use of liquid biopsy became the most frequently chosen option, accounting for 40 %, highlighting its crucial role as an alternative diagnostic tool when traditional tissue biopsies were not feasible. Finally, liquid biopsy played a role in monitoring treatment response, assisting physicians in evaluating the effectiveness of treatments and adjusting therapeutic plans as needed. In addition, physicians were more likely to opt for large gene panels in situations such as initial screening, inaccessibility of tissue samples, ineffective treatment or disease progression (Fig. 2). They also preferred large gene panels in situations where testing for TMB (tumor mutational burden) or MRD was required, or in consideration of patients' economic advantages.

### 3.2 The factors Influencing the willingness to adoption

**Fig. 3:**
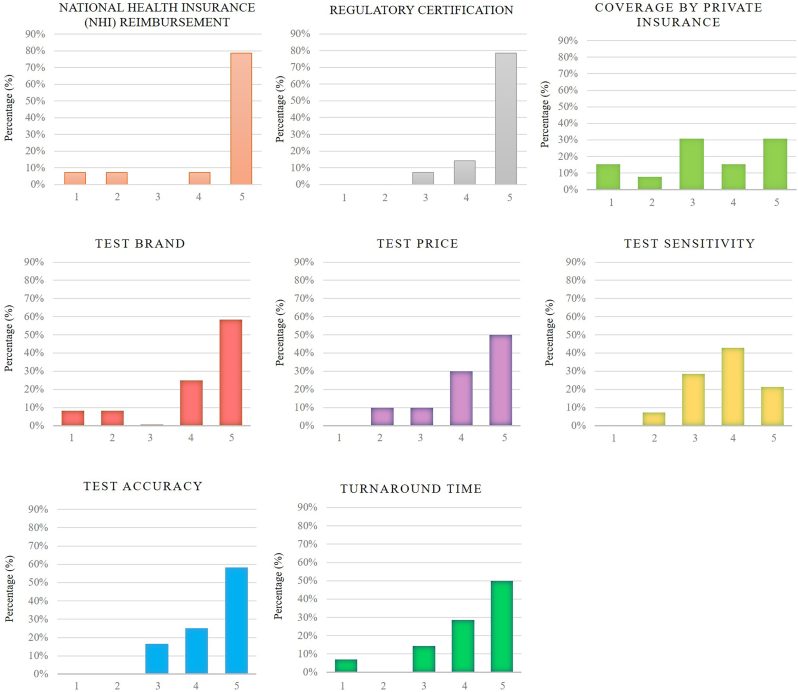
The questionnaire scores of the factors influencing willingness to adopt.

According to the participants, two main barriers were important to navigate in the translation of liquid biopsy in Taiwan, including NHI reimbursement policies and regulatory certification (Fig. 3). Clinicians generally considered National Health Insurance (NHI) reimbursement policies, with 79 % of respondents selecting a score of 5 (mean score = 4.4 ± 1.23) and regulatory certification (mean score = 4.7 ± 0.61) as the key factors influencing the clinical adoption of liquid biopsy. Currently, NHI reimbursement only covers tissue biopsy tests, which limits the use of liquid biopsy due to the lack of national reimbursement coverage. This limitation directly reduces physicians' willingness to incorporate liquid biopsy into their clinical practice in Taiwan. Regarding patients' private insurance claims, opinions differed across specialties, with 31 % of respondents selecting a score of 5 (mean score = 3.3 ± 1.34). The decision often depended on whether private insurance provided coverage. However, this did not appear to significantly influence physicians' willingness to advocate for liquid biopsy. In terms of test quality, specifically factors such as sensitivity, accuracy, price, and brand, physicians generally accepted that liquid biopsy had certain limitations in sensitivity, which did not substantially affect their willingness to adopt the technology. On the other hand, factors like accuracy, price, and brand were considered highly important, and they played a crucial role in motivating physicians to pursue liquid biopsy testing.

To evaluate the differences or similarities based on specialty and years of experience, physicians were grouped by specialty (thoracic medicine or hematologic oncology) and experience (more than 7 years or less than 7 years as attending physicians). According to the statistical results presented in Table 1, data showed that both thoracic medicine and hematologic oncology physicians had similar scores regarding NHI reimbursement for liquid biopsy testing, suggesting that both groups regarded it as a key factor influencing its usage. Additionally, both specialties shared similar views on the importance of regulatory certification and test accuracy, with these factors significantly impacting their willingness to adopt liquid biopsy. Physicians generally believed that once a test received regulatory certification, its accuracy was ensured. However, when it came to the selection of test brands, experienced physicians placed greater importance on the reputation and quality assurance of the brand, often recommending well-known brands to their patients. In contrast, younger physicians were more open to new brands, believing that the differences between testing companies were not substantial at present (senior physicians vs. junior physicians: 4.8 ± 0.37 vs. 3.3 ± 1.48; p value = 0.04) (Table 2).

**Table 1:** Influencing factor for adoption of liquid biopsy (By specialty).

	Hematologic oncology		Thoracic medicine		P-value
	Mean	SD	Mean	SD	
National Health Insurance (NHI) Reimbursement	4.4	1.05	4.4	1.40	0.99
Coverage by private insurance	3.5	1.38	3.3	1.39	0.80
Regulatory certification	4.6	0.69	4.8	0.97	0.55
Test sensitivity	4.0	0.71	3.5	0.96	0.32
Test accuracy	4.3	0.75	4.5	0.76	0.73
Test cost	3.8	1.07	4.2	1.07	0.63
The brand of the test	4.0	1.55	4.0	1.22	0.99
Turnaround time	3.7	1.28	4.6	0.73	0.18

**Table 2:** Influencing factor for adoption of liquid biopsy (By experience).

	Senior physicians ( ≥ 7yr)		Junior physicians (<7yr)		P-value
	Mean	SD	Mean	SD	
National Health Insurance (NHI) Reimbursement	4.1	1.54	4.8	0.37	0.33
Coverage by private insurance	3.7	1.50	3.1	1.25	0.54
Regulatory certification	4.9	0.33	4.5	0.76	0.27
Test sensitivity	3.9	0.78	3.7	0.94	0.68
Test accuracy	4.3	0.75	4.5	0.76	0.73
Test cost	3.8	1.07	1.2	1.07	0.63
**The brand of the test**	**4.8**	**0.37**	**3.3**	**1.48**	**0.04∗**
Turnaround time	3.7	1.28	4.6	0.73	0.18

### 3.3 Analysis of physician Groups and preferences

**Fig. 4:**
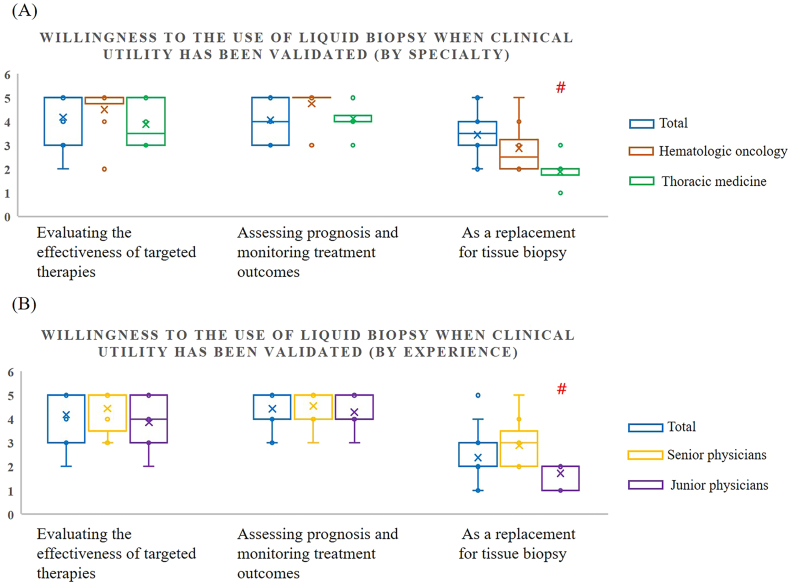
Willingness to the use of liquid biopsy by (A) specialty and (B) experience.

**Fig. 5:**
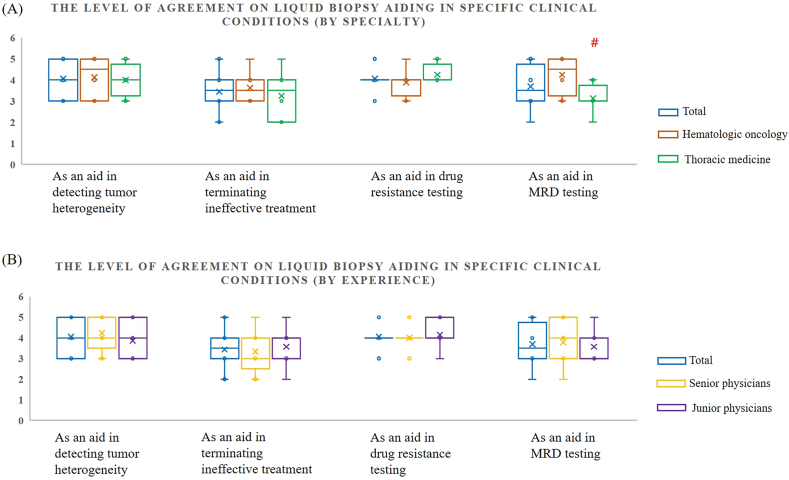
Level of agreement on liquid biopsy aiding in specific clinical conditions by (A) specialty and (B) experience.

We analyzed the differences in willingness to use liquid biopsy after clinical efficacy is validated, and its perceived usefulness in specific clinical conditions across specialties and experience levels. Generally, physicians agreed that liquid biopsy cannot fully replace tissue biopsy, which remains the clinical gold standard. This aligns with current practice in treating solid tumors, where liquid biopsy is more commonly used in later-line treatments for advanced-stage patients or when tissue samples are inaccessible. While neither hematologic oncologists nor thoracic medicine specialists were inclined to rely solely on liquid biopsy, hematologic oncologists were more open to the idea of using it as a replacement for tissue biopsy, especially once its clinical efficacy is validated (hematologic oncology vs. thoracic medicine: 2.9 ± 1.05 vs. 1.9 ± 0.60; p value = 0.04) (Fig. 4A and Table 3). This is closely tied to the nature of the patient population, as hematologic oncology patients are often in advanced stages of disease, which makes liquid biopsy a more viable option. Although many physicians were not yet fully convinced by the clinical evidence supporting liquid biopsy, as well as its limited use in Taiwan, experienced physicians tended to be more optimistic about its potential (senior physicians vs. junior physicians: 2.9 ± 1.00 vs. 1.7 ± 0.45; p value = 0.02) (Fig. 4B and Table 4). They were more open to considering it for preliminary testing, believing that technological advances would enhance its future utility. Regarding the advantages of liquid biopsy in specific clinical conditions, physicians generally agreed on its role in detecting tumor heterogeneity, assessing treatment effectiveness, testing for drug resistance, and monitoring MRD (Fig. 5). Hematologic oncologists demonstrated a significantly higher level of agreement with the use of liquid biopsy for MRD testing compared to thoracic medicine specialists (hematologic oncology vs. thoracic medicine: 4.2 ± 0.83 vs. 3.1 ± 0.60; p value = 0.01) (Fig. 5A and Table 3). However, there were no significant differences in agreement for the other conditions, nor was there any notable difference based on years of experience, with scores ranging from 3 to 4 (Fig. 5B and Table 4).

**Table 3:** Willingness to Use and Level of Agreement (By specialty).

	Hematologic oncology		Thoracic medicine		P-value
	Mean	SD	Mean	SD	
*Willingness to use*					
Evaluating the effectiveness of targeted therapies	4.5	1.00	3.9	0.93	0.25
Assessing prognosis and monitoring treatment outcomes	4.8	0.67	4.1	0.60	0.09
**As a replacement for tissue biopsy**	**2.9**	**1.05**	**1.9**	**0.60**	**0.04∗**
*Level of agreement*					
As an aid in detecting tumor heterogeneity	4.1	0.93	4.0	0.71	0.78
As an aid in terminating ineffective treatment	3.6	0.70	3.3	1.09	0.46
As an aid in drug resistance testing	3.9	0.60	4.3	0.43	0.20
**As an aid in MRD testing**	**4.2**	**0.83**	**3.1**	**0.60**	**0.01∗**

**Table 4:** Willingness to Use and Level of Agreement (By experience).

	Senior physicians ( ≥ 7yr)		Junior physicians (<7yr)		P-value
	Mean	SD	Mean	SD	
*Willingness to use*					
Evaluating the effectiveness of targeted therapies	4.4	0.83	3.9	1.12	0.28
Assessing prognosis and monitoring treatment outcomes	4.6	0.68	4.3	0.70	0.48
**As a replacement for tissue biopsy**	**2.9**	**1.00**	**1.7**	**0.45**	**0.02∗**
*Level of agreement*					
As an aid in detecting tumor heterogeneity	4.2	0.79	3.9	0.83	0.42
As an aid in terminating ineffective treatment	3.3	0.94	3.6	0.90	0.64
As an aid in drug resistance testing	4.0	0.47	4.1	0.64	0.64
**As an aid in MRD testing**	3.8	1.03	3.6	0.73	0.68

In summary, the clinical adoption of liquid biopsy is currently limited by several key factors, including National health insurance policies, test quality, and high costs. While liquid biopsy has the potential to benefit specific clinical scenarios, its broader implementation depends on the accumulation of robust clinical evidence and necessary policy changes. The primary barriers to wider use are the insufficient clinical data supporting its efficacy and the high testing costs, both of which make it difficult to encourage patients to undergo this diagnostic approach.

## 4 Discussion

Liquid biopsy is emerging as a valuable tool in cancer management, particularly for monitoring disease progression and detecting MRD. In this study, survey results indicate that factors influencing clinicians' willingness to adopt liquid biopsy include NHI reimbursement policies, regulatory approval, accuracy, and brand reputation. Currently in Taiwan, NHI only reimburses tissue biopsies, limiting the use of liquid biopsy. If liquid biopsy were included in NHI coverage, its adoption would likely increase. Analysis by specialty and experience showed that thoracic and hematology oncologists share similar views on regulatory certification, test quality, and costs, but differ in attitudes toward using liquid biopsy for MRD detection and as a substitute for tissue biopsies. Younger doctors tend to prioritize NHI reimbursement and report turnaround time, showing higher average scores in these areas, although the differences were not statistically significant. In contrast, more experienced physicians focus on brand reputation and quality assurance, with a more optimistic outlook on future potential of liquid biopsy.

Liquid biopsy faces several challenges in clinical practice in Taiwan, one of the primary barriers being the low mutation allele frequency (MAF) in circulating free DNA (cfDNA) [[22], [23], [24]]. Tissue biopsies typically have higher mutation frequencies due to the greater abundance of tumor cells [25], while liquid biopsy samples often exhibit MAFs ranging from 0.1 % to 5 %, necessitating precise thresholds for reliable interpretation [26]. This results in weak cancer mutation signals that are susceptible to interference from normal background DNA. Consequently, distinguishing clinically relevant mutations from background noise becomes a significant challenge, requiring highly sensitive detection methods to accurately capture low-frequency mutations. A study with 21,807 patients using a plasma DNA assay for genotyping advanced cancer found that the median MAF of true-positive variants was only 0.41 %, making it difficult to interpret low-frequency mutations as clinically relevant [27]. Recently, a study by Silveira et al. represented a significant advancement in overcoming the limitations of MAF in cfDNA detection [23]. By using ddPCR technique, they were able to detect and quantify the EGFR T790M mutation at much lower allele frequencies, as low as 0.5 %. This approach shows promise for more sensitive and early detection of mutations in clinical settings, particularly in cases with MRD or subtle tumor changes, thus addressing a major challenge in liquid biopsy [18]. However, the clinical significance of detecting mutations at such low frequencies, especially regarding treatment decisions and prognosis, still needs to be fully established. Despite the challenges posed by low mutation frequencies, liquid biopsy remains a valuable tool for early detection, disease monitoring, and guiding treatment strategies in oncology. Our research findings further highlight that physicians across different specialties share common concerns about the accuracy of liquid biopsy in mutation detection. A key challenge with tissue biopsies is the issue of insufficient sample availability, which is often addressed by liquid biopsy when tissue samples are inadequate. The survey results also revealed that, when tissue samples are insufficient, both hematologic oncologists and thoracic specialists tend to opt for medium-to large-scale liquid biopsy testing.

According to our research, hematologic oncologists are the primary practitioners who choose to request tumor mutational burden (TMB) and minimal residual disease (MRD) testing when opting for large gene panel-based liquid biopsy. As hematologic oncologists often liquid biopsy to assess immune-related biomarkers for immunotherapy eligibility. For MRD testing, liquid biopsy can detect trace amounts of ctDNA or CTCs, enabling early relapse detection not visible on traditional imaging. In contrast, the demand for MRD testing in lung cancer, particularly in NSCLC, is relatively low, as the disease is primarily monitored using imaging techniques (such as CT scans) and tumor markers (e.g., CEA and CYFRA 21–1). Furthermore, MRD testing in lung cancer is complicated by tumor heterogeneity, which can lead to inconsistencies in detecting ctDNA that represents minimal residual disease across different regions of the tumor. Consequently, while liquid biopsy is an effective tool for mutation detection and resistance monitoring in NSCLC, its application for MRD testing remains limited [28]. Moreover, the wide variety of ctDNA detection methods complicates the comparison of results across different studies. Although ctDNA can be useful for patient stratification, its impact on patient outcomes still requires further prospective trials, particularly among NSCLC patients undergoing biomarker-targeted treatments [29]. Increasing evidence suggests that MRD positivity in early-stage NSCLC could serve as a powerful negative prognostic indicator, as ctDNA testing before or after surgery, or following adjuvant treatments, is significantly associated with poorer outcomes [[30], [31], [32]]. Therefore, the integration of ctDNA data with other clinic-pathological information might increase the sensitivity of liquid biopsy for MRD detection and its informative value for perioperative therapies. While liquid biopsy has a clear advantage for MRD detection in hematologic malignancies, its application in lung cancer patients remains more limited, focusing on treatment response, mutation detection, and resistance monitoring.

The variation in ctDNA characteristics among cancer patients is influenced by tumor-specific factors, including tumor location, disease burden, proliferation and apoptosis rates, necrosis extent, inflammation, tumor microenvironment, and host-related phenomena. Choosing the appropriate type of ctDNA testing to address specific scientific and clinical questions is closely tied to the success of ctDNA analysis. At present, no single ctDNA test is suitable for all purposes, such as early detection, MRD analysis, gene variant identification, assessment of tumor genetic heterogeneity, and resistance detection, as well as the underlying molecular mechanisms of subsequent tumor progression. For early disease detection, patient-specific sequencing or methylation pattern-based methods may be needed for monitoring early-stage patients or conducting MRD analysis. In contrast, identifying treatment-resistant mutations and assessing tumor genetic heterogeneity in metastatic settings may require different types of tests. Various testing methods come with distinct limits of detection (LoD) and limits of quantification (LoQ), which ultimately determine the quantity of plasma DNA and ctDNA needed to yield reliable results [14,33,34]. Moreover, depending on the type of variation detected (such as copy number alterations (CNAs), somatic point mutations, epigenetic features, and fragmentomics) or the detection technologies used, ctDNA testing methods may be influenced by pre-analytical variables [11,12]. Several studies by individual investigators and consortia have sought to provide guidance on pre-analytical parameters and standard operating procedures (SOPs) to ensure consistency and reliability in ctDNA testing [35,36].

Although cfDNA NGS testing shows high concordance with tissue-based genotyping in large clinical studies, the proportion of biomarkers detected in cfDNA is still lower than that in the current tissue-based SOC. Therefore, cfDNA testing should primarily be reserved for reflex testing in cases where tissue samples are insufficient [37]. In a multi-center trial, Leighl et al. showed that a validated, sensitive cfDNA test in newly diagnosed metastatic NSCLC patients identified biomarkers recommended by clinical guidelines, with detection rates similar to SOC tissue-based genotyping [38]. Their findings suggested that initial biomarker assessment using cfDNA, plasma first instead of tissue, with tissue reserved for PD-L1 immunohistochemistry and reflex testing when cfDNA is negative for known oncogenic driver mutations, improves the biomarker discovery rate, TAT, and increases the number of newly diagnosed metastatic NSCLC patients receiving guideline-complete biomarker testing. These guideline-recommended biomarkers include *EGFR* mutations, *ALK* fusions, *ROS1* fusions, *BRAF* V600E mutation, *RET* fusions, *MET* amplification, *MET* exon 14 skipping variants, and *ERBB2* (HER2) mutations. Clinical practice guidelines have continued to expand, with the latest version of the NCCN guidelines advocating for the assessment of a ninth biomarker, *NTRK* fusions. These mutations vary in frequency across different populations, with some mutations, particularly *EGFR* and *ALK*, being more common in Asian populations [[39], [40], [41]]. The most common driver gene mutation in NSCLC is *EGFR*, which occurs in 45 % of Asian patients and 20 % of Caucasian patients with adenocarcinoma histology [42,43]. Therefore, liquid biopsy has the potential to accelerate treatment initiation and may improve the detection rate of actionable mutations, such as *EGFR* and *ALK*, which are more prevalent in East Asian patients.

However, the unique healthcare system in Taiwan, characterized by its universal coverage, presents its own set of barriers to the broader adoption of liquid biopsy. Taiwan's National Health Insurance (NHI) provides widely accessible and cost-effective medical services, enabling the majority of the population to easily obtain medical diagnoses and treatments [44]. Nevertheless, the system is confronted with several issues, including funding gaps, uneven distribution of healthcare resources, and overcrowded medical services. Due to the limited budget of the NHI system, the widespread adoption of innovative therapies and diagnostic tools is often constrained by financial limitations. Although liquid biopsy offers advantages such as being non-invasive, cost-effective, and enabling repeated monitoring, it still faces significant barriers to application, as it has not yet been fully incorporated into the NHI reimbursement scheme. [45]. Moreover, Taiwan's healthcare environment is predominantly outpatient-based, with heavy workloads for physicians and frequent patient visits. This may impact the willingness of healthcare providers to adopt new technologies such as liquid biopsy. According to current NHI reimbursement regulations, liquid biopsy remains in an experimental phase or is limited to certain applications, which may restrict its broader integration into routine clinical practice [46]. In clinical applications in Taiwan, liquid biopsy is often viewed as an addition to tissue biopsy, providing supplementary clinical and economic value. However, evidence for its cost-effectiveness remains limited, which may hinder its full implementation in NHI reimbursement and routine clinical practice. Lui et al. assessed various aspects, including policies, preferences, willingness to pay thresholds, and demographic characteristics, to evaluate the long-term cost-effectiveness of tissue versus liquid biopsy strategies in the Singaporean context [47]. Their findings suggested that sequential plasma-tissue NGS and plasma NGS alone were more costly and less effective than alternative approaches, whereas the sequential tissue-plasma NGS strategy generated the highest net monetary benefit. Therefore, the routine use of this approach in newly diagnosed advanced non-squamous NSCLC patients in Asian populations should be proactively considered. Additionally, Ezeife et al. reported that adding liquid biopsy to tissue biopsy could save CAD 3065 compared to tissue biopsy alone. This is because the combined approach can identify more patients eligible for targeted therapy, which is typically more cost-effective than chemotherapy or immunotherapy [48]. The potential benefit is especially pronounced in *EGFR* mutation-positive patients [49]. Despite these advantages, the tissue-first approach remains the best strategy for minimizing financial losses in current clinical practice.

The clinical validity of ctDNA testing has reached a level where sufficiently validated and sensitive ctDNA tests can be routinely used for advanced disease genotyping, provided the limitations of the test are well-understood and considered [14]. Increasing evidence showed that monitoring ctDNA levels in a series of plasma samples from metastatic cancer patients undergoing immune checkpoint inhibitor treatment can assess prognosis and therapeutic benefits. A pan-cancer analysis involving nearly 1000 patients with locally advanced/metastatic tumors treated with immune checkpoint blockade revealed that dynamic changes in ctDNA levels during treatment could predict potential long-term benefits of immune therapy across various tumor types [[50], [51], [52]]. Furthermore, ctDNA analysis enables the calculation of circulating tumor fraction (TF), which represents the proportion of tumor-derived DNA in plasma. TF is correlated with MAF and has demonstrated prognostic significance in several studies. While most prognostic models for metastatic cancers are tumor-type specific and require large patient-level datasets, the quantification of TF in ctDNA has been explored as a potential prognostic tool [53]. Additionally, TF dynamics may reflect pathological response, which is conventionally assessed using the residual cancer burden (RCB) score. Recently, Erve et al. used low-coverage whole genome sequencing to determine the cfDNA TF and validated in solid tumor patients. In patients with low tumor fraction scores during treatment, overall survival was longer, and the results were more accurate than imaging in predicting clinical outcomes [54]. Negative liquid biopsy with ctDNA TF <1 % commonly have a driver identified by follow-up tissue testing and should be prioritized for reflex testing. This approach can assist clinicians in deciding whether to act based on negative liquid biopsy results, initiate non-targeted treatments, or prioritize tissue acquisition for confirmatory comprehensive genomic analysis [55]. Another application of ctDNA TF is in treatment selection among several potential standard treatment options. For cancers like NSCLC, where immunotherapy and chemoimmunotherapy serve as alternative standard options, patients with favorable prognosis based on low TF and advantageous immunotherapy biomarkers could opt for immunotherapy alone to avoid chemotherapy toxicity. By combining TF with other liquid biopsy technologies such as NGS, RT-PCR, and ddPCR, the accuracy of early cancer detection may be improved, but further validation will be required.

Our findings highlight the significant potential of liquid biopsy, particularly in specialized testing areas, offering flexibility when covered treatments fail. For patients without available reimbursed options, medium-to large-scale liquid biopsy provides a valuable out-of-pocket alternative, eliminating the need for invasive procedures and broadening treatment choices, especially for personalized therapies. While liquid biopsy holds substantial promise in Taiwan, particularly in the self-pay market, its clinical use remains constrained by the lack of reimbursement under the current health insurance policies. To broaden its use, it is crucial for companies to collaborate with government and insurance stakeholders to advocate for policy reforms and facilitate the integration of liquid biopsy into reimbursement schemes. Additionally, conducting large-scale clinical trials to gather robust data on its clinical benefits is essential to demonstrate its value and encourage wider adoption. Further, there is a pressing need to increase the clinical implementation of liquid biopsies, particularly through ctDNA-based MRD detection, to enhance treatment monitoring and decision-making.

## 5 Limitation

This study faces several limitations in exploring the clinical application of liquid biopsy technology. Firstly, the limited number of surveyed physicians may result in insufficient representativeness of the findings. The imbalance in sample sizes could also affect the results of the data analysis. To enhance the significance and accuracy of the findings, future research should consider increasing the number of participating clinicians or conducting comparisons with an equal number of participants across groups, ensuring that statistical differences are meaningful. Additionally, the differences in group composition may impact the accuracy of the results. The majority of less experienced physicians were thoracic medicine specialists, while both hematologic oncologists and thoracic medicine specialists were equally represented among the more experienced physicians. Given that lung cancer, particularly NSCLC with EGFR mutations, is the most common malignancy among thoracic medicine patients and is covered by the NHI system in Taiwan, thoracic medicine specialists are therefore especially attentive to NHI reimbursement issues. In contrast, hematologic oncologists treat a more diverse range of cancers, many of which involve treatments that are less frequently reimbursed by the NHI system Taiwan, with most of the costs being self-paid. This difference in patient profiles could introduce bias in the data analysis, and such factors should be considered and adjusted for in future studies.

To address the limitations mentioned above, future research should aim to increase the sample size and ensure a balanced distribution of participants, including expanding the number of clinicians from various specialties. The inclusion of factors such as the type of medical institutions (e.g., regional hospitals, medical centers) could further enhance the representativeness and significance of the findings. Additionally, stratified analysis based on years of experience and specialty should be conducted to explore differences in the adoption of liquid biopsy technology across various groups, thereby minimizing biases arising from differences in group composition. Future studies should also examine how clinicians from different cancer specialties perceive the significance of NHI reimbursement, particularly regarding the impact of liquid biopsy technology on reimbursement applications. Longitudinal studies should be conducted to assess the actual clinical impact of liquid biopsy technology, providing empirical evidence of its benefits across various clinical contexts. These approaches will enable a more comprehensive exploration of the clinical value of liquid biopsy technology in future research.

## 6 Conclusion

In conclusion, liquid biopsy shows significant promise as a non-invasive tool for the diagnosis and monitoring of solid tumors and hematologic cancers, particularly in the context of MRD. The survey results highlight that liquid biopsy is highly valued in clinical stages such as initial diagnosis, disease monitoring, and treatment response evaluation, especially when tissue biopsies are not feasible. However, its broader application in Taiwan is currently limited by NHI reimbursement policies, which only cover tissue biopsy tests, as well as regulatory certification hurdles. These barriers significantly influence clinicians' willingness to adopt liquid biopsy in routine practice.

In Taiwan's healthcare system, the NHI system remains a critical factor in determining the adoption of emerging diagnostic technologies like liquid biopsy. Currently, liquid biopsy testing is not reimbursed for routine clinical use, which limits its potential in detecting MRD and monitoring disease recurrence. Both thoracic medicine specialists and hematologic oncologists recognize the importance of NHI reimbursement and regulatory approval in driving liquid biopsy adoption. While hematologic oncologists, who often treat advanced cancers, are more open to replacing tissue biopsy with liquid biopsy, thoracic medicine specialists are more cautious. Moreover, experienced physicians are generally more optimistic about liquid biopsy's future, particularly as its clinical efficacy is demonstrated.

To fully realize the potential of liquid biopsy in clinical practice, especially for MRD detection in cancers such as lung and hematologic malignancies, future research should focus on generating robust clinical data to support its effectiveness. Additionally, advocacy for policy changes to include liquid biopsy in the NHI reimbursement system is crucial. With improvements in diagnostic technology, cost reduction, and policy reform, liquid biopsy could become an integral tool in personalized cancer care in Taiwan, offering more accurate and timely monitoring of treatment response and recurrence.

## Informed consent statement

Not applicable.

## Author contributions

Conceptualization, SH Chang and HY Li; Methodology, SH Chang and CC Chang; Formal analysis, CC Chang and CY Yao; Writing-original draft preparation, SH Chang, JCH Hsieh and YH Yang; Writing-review and editing, HY Li; Supervision, HY Li and SH Chang. All authors have read and agreed to the published version of the manuscript.

## Data availability statement

The datasets used and/or analyzed during the current study are available from the corresponding author on reasonable request.

## Institutional review board statement

Not applicable.

## Funding

No.

## Declaration of competing interest

The authors declare that they have no known competing financial interests or personal relationships that could have appeared to influence the work reported in this paper.

## References

[1] Bidard F.C., Michiels S., Riethdorf S., Mueller V., Esserman L.J., Lucci A., Naume B., Horiguchi J., Gisbert-Criado R., Sleijfer S. (2018). Circulating tumor cells in breast cancer patients treated by neoadjuvant chemotherapy: a meta-analysis. J Natl Cancer Inst.

[2] Hindson B.J., Ness K.D., Masquelier D.A., Belgrader P., Heredia N.J., Makarewicz A.J., Bright I.J., Lucero M.Y., Hiddessen A.L., Legler T.C. (2011). High-throughput droplet digital PCR system for absolute quantitation of DNA copy number. Anal Chem.

[3] Underwood J.J., Quadri R.S., Kalva S.P., Shah H., Sanjeevaiah A.R., Beg M.S., Sutphin P.D. (2020). Liquid biopsy for cancer: review and implications for the radiologist. Radiology.

[4] Aceto N., Bardia A., Miyamoto D.T., Donaldson M.C., Wittner B.S., Spencer J.A., Yu M., Pely A., Engstrom A., Zhu H. (2014). Circulating tumor cell clusters are oligoclonal precursors of breast cancer metastasis. Cell.

[5] Jung A., Kirchner T. (2018). Liquid biopsy in tumor genetic diagnosis. Dtsch Arztebl Int.

[6] Diehl F., Schmidt K., Choti M.A., Romans K., Goodman S., Li M., Thornton K., Agrawal N., Sokoll L., Szabo S.A. (2008). Circulating mutant DNA to assess tumor dynamics. Nat Med.

[7] de Bono J.S., Scher H.I., Montgomery R.B., Parker C., Miller M.C., Tissing H., Doyle G.V., Terstappen L.W., Pienta K.J., Raghavan D. (2008). Circulating tumor cells predict survival benefit from treatment in metastatic castration-resistant prostate cancer. Clin Cancer Res.

[8] Pantel K., Alix-Panabieres C. (2010). Circulating tumour cells in cancer patients: challenges and perspectives. Trends Mol Med.

[9] Duffy M.J. (2024). Circulating tumor DNA (ctDNA) as a biomarker for lung cancer: early detection, monitoring and therapy prediction. Tumour Biol.

[10] Oshiro C., Kagara N., Naoi Y., Shimoda M., Shimomura A., Maruyama N., Shimazu K., Kim S.J., Noguchi S. (2015). PIK3CA mutations in serum DNA are predictive of recurrence in primary breast cancer patients. Breast Cancer Res Treat.

[11] Ma L., Guo H., Zhao Y., Liu Z., Wang C., Bu J., Sun T., Wei J. (2024). Liquid biopsy in cancer current: status, challenges and future prospects. Signal Transduct Target Ther.

[12] Rolfo C., Cardona A.F., Cristofanilli M., Paz-Ares L., Diaz Mochon J.J., Duran I., Raez L.E., Russo A., Lorente J.A., Malapelle U. (2020). Corrigendum to “challenges and opportunities of cfDNA analysis implementation in clinical practice: perspective of the international society of liquid biopsy (ISLB)”. Crit Rev Oncol Hematol.

[13] Rolfo C., Mack P., Scagliotti G.V., Aggarwal C., Arcila M.E., Barlesi F., Bivona T., Diehn M., Dive C., Dziadziuszko R. (2021). Liquid biopsy for advanced NSCLC: a consensus statement from the international association for the study of lung cancer. J Thorac Oncol.

[14] Pascual J., Attard G., Bidard F.C., Curigliano G., De Mattos-Arruda L., Diehn M., Italiano A., Lindberg J., Merker J.D., Montagut C. (2022). ESMO recommendations on the use of circulating tumour DNA assays for patients with cancer: a report from the ESMO Precision Medicine Working Group. Ann Oncol.

[15] Gorges T.M., Pantel K. (2013). Circulating tumor cells as therapy-related biomarkers in cancer patients. Cancer Immunol Immunother.

[16] Abbosh C., Birkbak N.J., Wilson G.A., Jamal-Hanjani M., Constantin T., Salari R., Le Quesne J., Moore D.A., Veeriah S., Rosenthal R. (2017). Phylogenetic ctDNA analysis depicts early-stage lung cancer evolution. Nature.

[17] Nakamura Y., Tsukada Y., Matsuhashi N., Murano T., Shiozawa M., Takahashi Y., Oki E., Goto M., Kagawa Y., Kanazawa A. (2024). Colorectal cancer recurrence prediction using a tissue-free epigenomic minimal residual disease assay. Clin Cancer Res.

[18] Chen K., Shields M.D., Chauhan P.S., Ramirez R.J., Harris P.K., Reimers M.A., Zevallos J.P., Davis A.A., Pellini B., Chaudhuri A.A. (2021). Commercial ctDNA assays for minimal residual disease detection of solid tumors. Mol Diagn Ther.

[19] Cristofanilli M., Hayes D.F., Budd G.T., Ellis M.J., Stopeck A., Reuben J.M., Doyle G.V., Matera J., Allard W.J., Miller M.C. (2005). Circulating tumor cells: a novel prognostic factor for newly diagnosed metastatic breast cancer. J Clin Oncol.

[20] Gerlinger M., Rowan A.J., Horswell S., Math M., Larkin J., Endesfelder D., Gronroos E., Martinez P., Matthews N., Stewart A. (2012). Intratumor heterogeneity and branched evolution revealed by multiregion sequencing. N Engl J Med.

[21] Wei T., Zhang Q., Li X., Su W., Li G., Ma T., Gao S., Lou J., Que R., Zheng L. (2019). Monitoring tumor burden in response to FOLFIRINOX chemotherapy via profiling circulating cell-free DNA in pancreatic cancer. Mol Cancer Ther.

[22] Mok T., Wu Y.L., Lee J.S., Yu C.J., Sriuranpong V., Sandoval-Tan J., Ladrera G., Thongprasert S., Srimuninnimit V., Liao M. (2015). Detection and dynamic changes of EGFR mutations from circulating tumor DNA as a predictor of survival outcomes in NSCLC patients treated with first-line intercalated erlotinib and chemotherapy. Clin Cancer Res.

[23] Silveira C., Sousa A.C., Janeiro A., Malveiro S., Teixeira E., Brysch E., Pantarotto M., Felizardo M., Madureira R., Nogueira F. (2021). Detection and quantification of EGFR T790M mutation in liquid biopsies by droplet digital PCR. Transl Lung Cancer Res.

[24] Abbosh C., Birkbak N.J., Swanton C. (2018). Early stage NSCLC - challenges to implementing ctDNA-based screening and MRD detection. Nat Rev Clin Oncol.

[25] Kinde I., Wu J., Papadopoulos N., Kinzler K.W., Vogelstein B. (2011). Detection and quantification of rare mutations with massively parallel sequencing. Proc Natl Acad Sci U S A.

[26] Dudley J.C., Diehn M. (2021). Detection and diagnostic utilization of cellular and cell-free tumor DNA. Annu Rev Pathol.

[27] Zill O.A., Banks K.C., Fairclough S.R., Mortimer S.A., Vowles J.V., Mokhtari R., Gandara D.R., Mack P.C., Odegaard J.I., Nagy R.J. (2018). The landscape of actionable genomic alterations in cell-free circulating tumor DNA from 21,807 advanced cancer patients. Clin Cancer Res.

[28] Planchard D., Popat S., Kerr K., Novello S., Smit E.F., Faivre-Finn C., Mok T.S., Reck M., Van Schil P.E., Hellmann M.D. (2018). Metastatic non-small cell lung cancer: ESMO Clinical Practice Guidelines for diagnosis, treatment and follow-up. Ann Oncol.

[29] Passarella G., Canova S., Abbate M.I., Caspani G., Sala L., Russo A., Muscolino P., Colonese F., Cortinovis D.L. (2025). The hype around ctDNA guiding an informed perioperative therapeutic strategy in early-stage non-small cell lung cancer. Discov Oncol.

[30] Forde P.M., Spicer J., Lu S., Provencio M., Mitsudomi T., Awad M.M., Felip E., Broderick S.R., Brahmer J.R., Swanson S.J. (2022). Neoadjuvant nivolumab plus chemotherapy in resectable lung cancer. N Engl J Med.

[31] Tran H.T., Heeke S., Sujit S., Vokes N., Zhang J., Aminu M., Lam V.K., Vaporciyan A., Swisher S.G., Godoy M.C.B. (2024). Circulating tumor DNA and radiological tumor volume identify patients at risk for relapse with resected, early-stage non-small-cell lung cancer. Ann Oncol.

[32] Felip E., Altorki N., Zhou C., Csoszi T., Vynnychenko I., Goloborodko O., Luft A., Akopov A., Martinez-Marti A., Kenmotsu H. (2021). Adjuvant atezolizumab after adjuvant chemotherapy in resected stage IB-IIIA non-small-cell lung cancer (IMpower010): a randomised, multicentre, open-label, phase 3 trial. Lancet.

[33] Parpart-Li S., Bartlett B., Popoli M., Adleff V., Tucker L., Steinberg R., Georgiadis A., Phallen J., Brahmer J., Azad N. (2017). The effect of preservative and temperature on the analysis of circulating tumor DNA. Clin Cancer Res.

[34] Markus H., Contente-Cuomo T., Farooq M., Liang W.S., Borad M.J., Sivakumar S., Gollins S., Tran N.L., Dhruv H.D., Berens M.E. (2018). Evaluation of pre-analytical factors affecting plasma DNA analysis. Sci Rep.

[35] Lampignano R., Neumann M.H.D., Weber S., Kloten V., Herdean A., Voss T., Groelz D., Babayan A., Tibbesma M., Schlumpberger M. (2020). Multicenter evaluation of circulating cell-free DNA extraction and downstream analyses for the development of standardized (Pre)analytical work flows. Clin Chem.

[36] Connors D., Allen J., Alvarez J.D., Boyle J., Cristofanilli M., Hiller C., Keating S., Kelloff G., Leiman L., McCormack R. (2020). International liquid biopsy standardization alliance white paper. Crit Rev Oncol Hematol.

[37] Russo A., Lee J.K., Pasquina L.W., Del Re M., Dilks H.H., Murugesan K., Madison R.W., Lee Y., Schrock A.B., Comment L. (2024). Liquid biopsy of lung cancer before pathological diagnosis is associated with shorter time to treatment. JCO Precis Oncol.

[38] Leighl N.B., Page R.D., Raymond V.M., Daniel D.B., Divers S.G., Reckamp K.L., Villalona-Calero M.A., Dix D., Odegaard J.I., Lanman R.B. (2019). Clinical utility of comprehensive cell-free DNA analysis to identify genomic biomarkers in patients with newly diagnosed metastatic non-small cell lung cancer. Clin Cancer Res.

[39] Han B., Tjulandin S., Hagiwara K., Normanno N., Wulandari L., Laktionov K., Hudoyo A., He Y., Zhang Y.P., Wang M.Z. (2017). EGFR mutation prevalence in Asia-Pacific and Russian patients with advanced NSCLC of adenocarcinoma and non-adenocarcinoma histology: the IGNITE study. Lung Cancer.

[40] Shaw A.T., Yeap B.Y., Mino-Kenudson M., Digumarthy S.R., Costa D.B., Heist R.S., Solomon B., Stubbs H., Admane S., McDermott U. (2009). Clinical features and outcome of patients with non-small-cell lung cancer who harbor EML4-ALK. J Clin Oncol.

[41] Lin C.W., Huang K.Y., Lin C.H., Hou M.H., Lin S.H. (2025). Diverse clinical outcomes for the EGFR-mutated and ALK-rearranged advanced non-squamous non-small cell lung cancer. Oncol Lett.

[42] Kim E.S., Melosky B., Park K., Yamamoto N., Yang J.C. (2021). EGFR tyrosine kinase inhibitors for EGFR mutation-positive non-small-cell lung cancer: outcomes in Asian populations. Future Oncol.

[43] Hu H., Tan S., Xie M., Guo P., Yu Q., Xiao J., Zhao K., Liao Q., Wang Y. (2023). Case report: concomitant EGFR mutation and ALK rearrangement in non-small cell lung cancer. Front Pharmacol.

[44] Yang S.W., Chu K.C., Kreng V.B. (2021). The impact of global budgeting on the efficiency of healthcare under a single-payer system in taiwan. Int J Environ Res Public Health.

[45] Wu T.Y., Majeed A., Kuo K.N. (2010). An overview of the healthcare system in Taiwan. London J Prim Care (Abingdon).

[46] Reinhardt U.E. (2008). Humbled in taiwan. BMJ.

[47] Liu S., Graves N., Tan A.C. (2024). The cost-effectiveness of including liquid biopsy into molecular profiling strategies for newly diagnosed advanced non-squamous non-small cell lung cancer in an Asian population. Lung Cancer.

[48] Ezeife D.A., Spackman E., Juergens R.A., Laskin J.J., Agulnik J.S., Hao D., Laurie S.A., Law J.H., Le L.W., Kiedrowski L.A. (2022). The economic value of liquid biopsy for genomic profiling in advanced non-small cell lung cancer. Ther Adv Med Oncol.

[49] Englmeier F., Bleckmann A., Bruckl W., Griesinger F., Fleitz A., Nagels K. (2023). Clinical benefit and cost-effectiveness analysis of liquid biopsy application in patients with advanced non-small cell lung cancer (NSCLC): a modelling approach. J Cancer Res Clin Oncol.

[50] Zhang Q., Luo J., Wu S., Si H., Gao C., Xu W., Abdullah S.E., Higgs B.W., Dennis P.A., van der Heijden M.S. (2020). Prognostic and predictive impact of circulating tumor DNA in patients with advanced cancers treated with immune checkpoint blockade. Cancer Discov.

[51] Cabel L., Riva F., Servois V., Livartowski A., Daniel C., Rampanou A., Lantz O., Romano E., Milder M., Buecher B. (2017). Circulating tumor DNA changes for early monitoring of anti-PD1 immunotherapy: a proof-of-concept study. Ann Oncol.

[52] Bratman S.V., Yang S.Y.C., Iafolla M.A.J., Liu Z., Hansen A.R., Bedard P.L., Lheureux S., Spreafico A., Razak A.A., Shchegrova S. (2020). Personalized circulating tumor DNA analysis as a predictive biomarker in solid tumor patients treated with pembrolizumab. Nat Cancer.

[53] Reichert Z.R., Morgan T.M., Li G., Castellanos E., Snow T., Dall'Olio F.G., Madison R.W., Fine A.D., Oxnard G.R., Graf R.P. (2023). Prognostic value of plasma circulating tumor DNA fraction across four common cancer types: a real-world outcomes study. Ann Oncol.

[54] van 't Erve I., Alipanahi B., Lumbard K., Skidmore Z.L., Rinaldi L., Millberg L.K., Carey J., Chesnick B., Cristiano S., Portwood C. (2024). Cancer treatment monitoring using cell-free DNA fragmentomes. Nat Commun.

[55] Rolfo C.D., Madison R.W., Pasquina L.W., Brown D.W., Huang Y., Hughes J.D., Graf R.P., Oxnard G.R., Husain H. (2024). Measurement of ctDNA tumor fraction identifies informative negative liquid biopsy results and informs value of tissue confirmation. Clin Cancer Res.

