# Supplementary material for: The prospects and limitations of liquid biopsy utilization for clinical practice in Taiwan
Source: J Liq Biopsy. 2025 Mar 6;7:100290. doi: 10.1016/j.jlb.2025.100290 (PMC11937291; doi:10.1016/j.jlb.2025.100290)
Supplement: Multimedia component 1 [file mmc1.docx]

| Question No. | Question | Hematology Oncology Physicians | Thoracic Medicine Physicians |
| --- | --- | --- | --- |
| 1 | When do you think liquid biopsy should be used in lymphoma/NSCLC patients? | □ Early detection,  □ 1st-line treatment,  □ 2nd-line or disease progression,  □ Difficult to obtain tissue biopsy,  □ Monitoring disease response | □ Early detection,  □ 1st-line treatment,  □ 2nd-line or disease progression,  □ Difficult to obtain tissue biopsy,  □ Monitoring disease response |
| 2 | What factors influence your willingness to use liquid biopsy in lymphoma/NSCLC? | □ NHI reimbursement via liquid biopsy (1-5 scale),  □ Coverage by private insurance (1-5 scale),  □ Regulatory certification (LDTs, IVD) (1-5 scale),  □ Sensitivity in specific contexts (e.g., MRD) (1-5 scale),  □ Accuracy (1-5 scale),  □ Test price (1-5 scale),  □ Test brand (1-5 scale),  □ Turnaround time (TAT) (1-5 scale) | □ NHI reimbursement via liquid biopsy (1-5 scale),  □ Coverage by private insurance (1-5 scale),  □ Regulatory certification (LDTs, IVD) (1-5 scale),  □ Sensitivity in specific contexts (e.g., MRD) (1-5 scale),  □ Accuracy (1-5 scale),  □ Test price (1-5 scale),  □ Test brand (1-5 scale),  □ Turnaround time (TAT) (1-5 scale) |
| 3 | Will you use liquid biopsy to evaluate lymphoma/NSCLC treatment efficacy (specifically for EGFR T790M mutations) compared to blood smear/tissue biopsy? | □ Very unwilling,  □ Unwilling,  □ Neutral,  □ Willing,  □ Very willing | □ Very unwilling,  □ Unwilling,  □ Neutral,  □ Willing,  □ Very willing |
| 4 | Will you use liquid biopsy for assessing prognosis or monitoring treatment outcomes compared to blood smear/tissue biopsy? | □ Very unwilling,  □ Unwilling,  □ Neutral,  □ Willing,  □ Very willing | □ Very unwilling,  □ Unwilling,  □ Neutral,  □ Willing,  □ Very willing |
| 5 | Would you be willing to replace blood smear/tissue biopsy with liquid biopsy for lymphoma/NSCLC evaluation if clinical efficacy is proven? | □ Very unwilling,  □ Unwilling,  □ Neutral,  □ Willing,  □ Very willing | □ Very unwilling,  □ Unwilling,  □ Neutral,  □ Willing,  □ Very willing |
| 6 | Do you agree that liquid biopsy can improve detection of tumor heterogeneity in lymphoma/NSCLC? | □ Strongly disagree,  □ Disagree,  □ Neutral,  □ Agree,  □ Strongly agree | □ Strongly disagree,  □ Disagree,  □ Neutral,  □ Agree,  □ Strongly agree |
| 7 | Do you agree that liquid biopsy can monitor treatment response and allow early cessation of ineffective therapies for non-solid tumors? | □ Strongly disagree,  □ Disagree,  □ Neutral,  □ Agree,  □ Strongly agree | □ Strongly disagree,  □ Disagree,  □ Neutral,  □ Agree,  □ Strongly agree |
| 8 | Do you agree that liquid biopsy is more useful for detecting drug resistance in metastatic non-solid tumors? | □ Strongly disagree,  □ Disagree,  □ Neutral,  □ Agree,  □ Strongly agree | □ Strongly disagree,  □ Disagree,  □ Neutral,  □ Agree,  □ Strongly agree |
| 9 | Do you agree that liquid biopsy is useful for detecting minimal residual disease (MRD) in lymphoma/NSCLC? | □ Strongly disagree,  □ Disagree,  □ Neutral,  □ Agree,  □ Strongly agree | □ Strongly disagree,  □ Disagree,  □ Neutral,  □ Agree,  □ Strongly agree |
| 10 | Under what conditions would you choose to use a large gene panel (>100 genes) for liquid biopsy in lymphoma/NSCLC? | [Open-ended response] | [Open-ended response] |
